# Morphogenesis of Aragonite Biomineral Structures by the Nonclassical Colloidal Crystal Growth Mechanism Revisited on the Nanoscale: The Noah’s Ark Shell (*Arca noae*, L.) Case Study

**DOI:** 10.1021/acsbiomaterials.4c01420

**Published:** 2025-01-29

**Authors:** Ivan Sondi, Adrijana Leonardi, Igor Križaj, Saša Kazazić, Branka Salopek-Sondi, Srečo D. Škapin

**Affiliations:** † Faculty of Mining, Geology and Petroleum Engineering, 10000 Zagreb, Croatia; ‡ Department of Molecular and Biomedical Sciences, Jožef Stefan Institute, 1000 Ljubljana, Slovenia; § Division of Physical Chemistry, Rud̵er Bošković Institute, 10000 Zagreb, Croatia; ∥ Division of Molecular Biology, Rud̵er Bošković Institute, 10000 Zagreb, Croatia; ⊥ Advanced Materials Department, Jožef Stefan Institute, 1000 Ljubljana, Slovenia

**Keywords:** aragonite, biomineralization, colloidally mediated mechanism, dissolution and recrystallization processes, mollusks

## Abstract

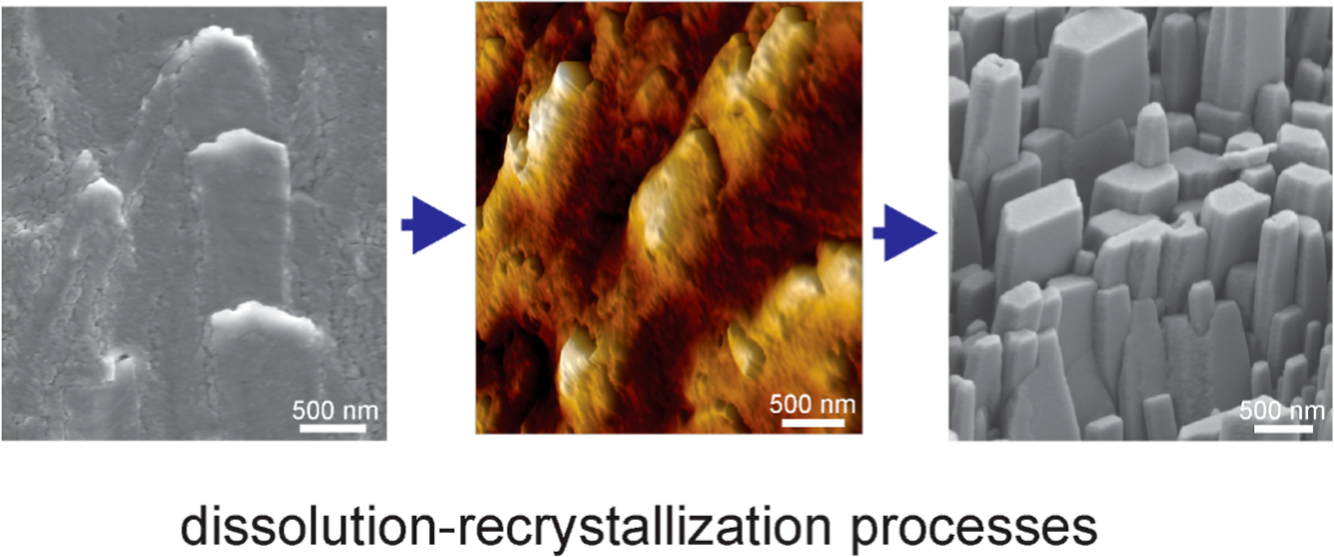

Characterization and formation of the biomineral aragonite structures of the Noah’s Ark shell (*Arca noae* L.,1758) were studied from structural, morphogenetic, and biochemical points of view. Structural and morphological features were examined using X-ray diffraction, field-emission scanning electron microscopy, and atomic force microscopy, while thermal properties were determined by thermogravimetric and differential thermal analyses. Proteins from the soluble organic matrix (SOM) were analyzed by Edman degradation. The results showed that the Noah’s Ark shell exhibits several distinct biomineral structures characterized by complex morphologies and different forms of aragonite. The inner shell of the Ark is characterized by a combination of nanogranular surfaces and micron-sized, idiomorphically developed aragonite crystals indicative of orthorhombic symmetry. The formation of these structures is discussed in terms of the nonclassical crystal growth route considering the colloidally mediated mechanism based on the initial particle–particle interaction of the nanosized and metastable precursor aragonite phase and their dissolution and recrystallization processes. These structures contained a small amount of connecting organic material, SOM, assessed at 1.5% of the total mass. Edman degradation revealed the partial amino acid sequence that is present also in the tetratricopeptide repeat (TPR) protein 8 from diverse mussels. Bacterial TPR-containing protein was found to be involved in the biomineralization process, so we propose such a function for these proteins also in mussels.

## 1 Introduction

Biominerals are the natural composite structures found in many organisms, characterized by a variety of hierarchically organized forms and complex shapes composed of different mineral phases and organic compounds.^1−3^ For several decades, much attention has been paid to the study of the basic mechanisms involved in the formation of biominerals belonging to the group of calcium carbonates. From this group, aragonite, the orthorhombic polymorphic modification of anhydrous calcium carbonate, CaCO_3_ (space group Pcmn), has certainly attracted the most attention, since many mineralizing organisms produce their biomineral structures from this mineral.^3−12^ Today, it is generally thought that most biomineral structures are formed by colloidally mediated crystal growth mechanisms, which primarily involve the attachment of particles, leading to the formation of bridged nanocrystals, aligned nanostructures, and mesocrystalline assemblies.^13−16^ These mechanisms have recently been recognized in the formation of biomineral structures in early animals, and it appears that they have not been altered during the evolutionary development of organisms over hundreds of millions of years.^14,17^ Several studies reported the role of nonclassical growth mechanisms in the formation of complex biomineral structures from aragonite.^5,11,18−21^ Undoubtedly, these processes are controlled by a specific organic matrix, a complex mixture of various proteins, polysaccharides, and traces of lipids and pigments.^22,23^ Mineralizing organisms utilize the capabilities of these compounds to control the initial nucleation of mineral phases and growth and formation of complex biomineral structures through their specific interaction with the surfaces of growing crystals.^1,2,24^ Our previous research confirmed this concept and showed that the general strategy for the morphogenesis of fibrous structured aragonite in the Scleractinian coral *Cladocora caespitosa*(9) and the biomineral structures of *Sepia officinalis*(4) lies in the so-called “bottom-up morphogenesis” based on the simultaneous nanoscale-oriented aggregation and subsequent coalescence processes of primarily formed aragonite nanogranules. Many mineralizing mollusks generate their biomineral structures from crossed lamellar carbonate assemblages.^7,18,24−26^ The Noah’s Ark (*Arca noae*, L.) shell has a well-organized biomineral form constructed from the combination of crossed lamellar and complex crossed lamellar (CCL) aragonite,^24,27,28^ in the form of a thick trapezoidal shape with distinct ribs on the outer surface. The biomineral structures of the aragonite in this species have been described at the micron-sized level.^27^

To the best of our knowledge, there are no recent comprehensive studies describing the morphological and structural characteristics of the aragonite biomineral structure of the Noah’s Ark shell at the submicron level. Moreover, the morphological and structural characteristics and formation of the biomineral structures occurring on the inner growth surface of the Noah’s Ark shell have not been adequately documented in the literature. Reports on the characteristics of the soluble organic matrix (SOM), especially proteins involved in the biomineralization processes of this species, are scarce.

Our structural and morphological characterization of different aragonite forms occurring on the inner surface and in a cross section of the Noah’s Ark shell and the study of the biomineralization process in this mollusk therefore fill these gaps and contribute to the knowledge of the formation of complex biomineral structures of aragonite attained in other species.^7,18,24,25^

## 2 Experimental Section

### 2.1 Chemicals

The reagent-grade chemicals used in this study were used without further purification. Stock solutions of all reactants were freshly prepared and filtered through 0.22 μm Millipore membranes before use.

### 2.2 Noah’s Ark Mussel—Sampling and Preparation of Its Shell

The shell hinge of Noah’s Ark is considered representative of an ancient ancestor because of its plesiomorphic feature—a taxodont dentition consisting of a series of individual homodont teeth extending over long hinge edges.^29^ The biomineral structure of the Noah’s Ark shell has been described as one of the most geometrically complex and compact biomineral forms of mollusks, containing a small amount of organic matrix and exhibiting excellent mechanical performance and great resistance to bending and fracturing.^30,31^

Noah’s Ark mussels (*A. noae*, L.) were sampled by scuba diving in the Adriatic Sea Mljet Island (Adriatic Sea) and were immediately stored on ice. After removing organisms, shells were mechanically scrubbed to remove all impurities and washed with Milli-Q water (Millipore Corporation, USA). Shells were then stored at 4 °C until analysis. This treatment was thorough enough to remove all of impurities. Shells of adult mussels with a size of 6–7 cm were chosen for analysis as the most representative specimens.

### 2.3 Methods

#### 2.3.1 Structural, Chemical, and Morphological Characterization of the Noah’s Ark Shell

The mineral composition of the Noah’s Ark shell was analyzed by powder X-ray diffraction (XRD), using a diffractometer with CuKα radiation and a Sol-X energy-dispersive detector (D4 Endeavor, Bruker AXS, Karlsruhe, Germany). The angular range 2Θ was from 10 to 70° with a step size of 0.02° and a collection time of 5 s. The obtained XRD patterns were identified in accordance with the ICDD powder-diffraction files.

The morphologies of the bioinorganic structures from the Noah’s Ark shell were examined using field-emission scanning electron microscopy (FESEM Zeiss, Ultra plus, Germany). Cleaned Noah’s Ark shell samples were carbon-coated using a PECS (Gatan, Model 682, Germany) to ensure good conductivity during the FESEM investigation. The cross-section of the shell fracture was analyzed using a stereo microscope (Discovery V8, Zeiss). The three-layered structure is presented in Figure S1.

**Figure 1:**
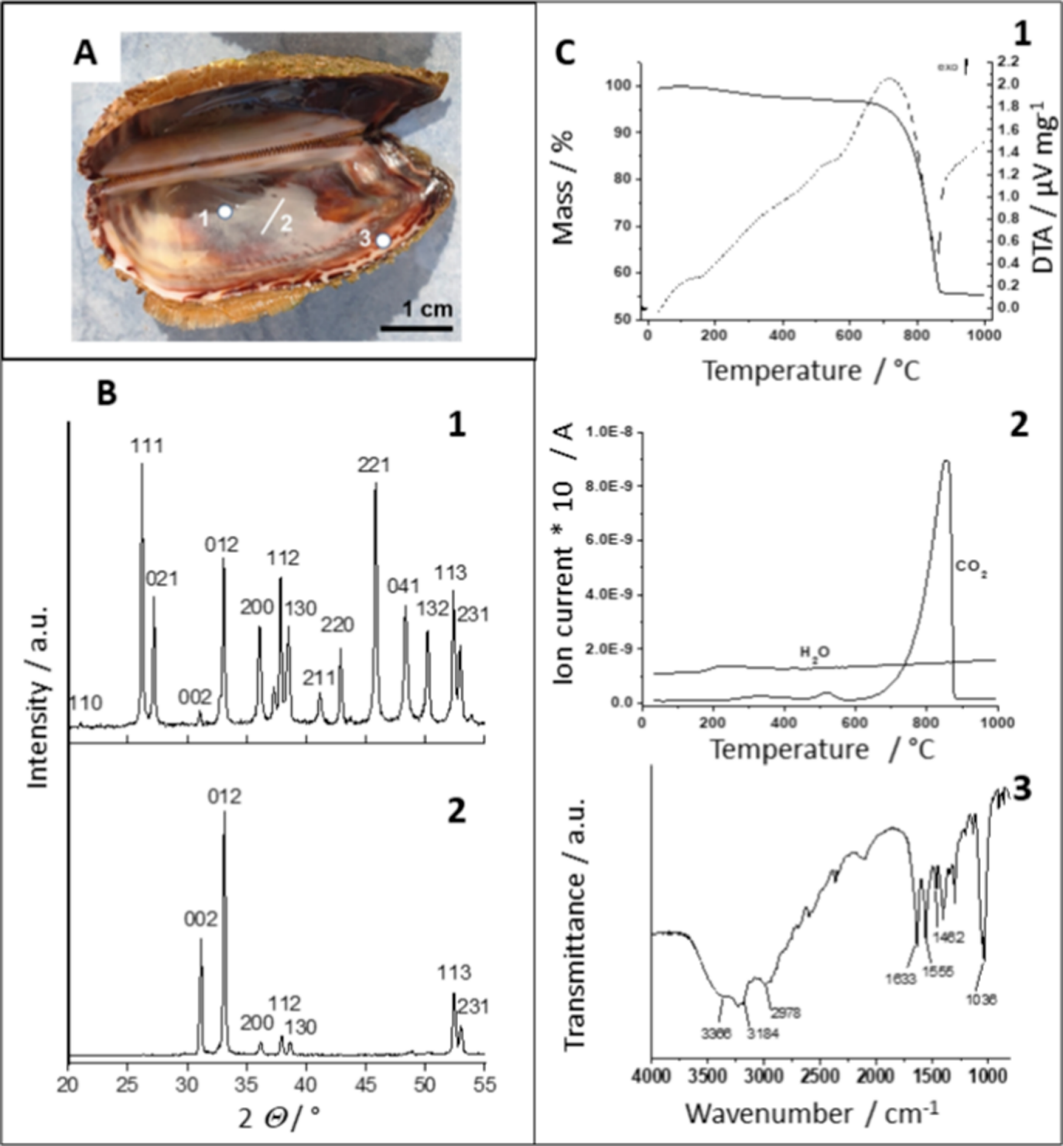
Noah’s Ark shell used in our experiments. (A) Positions where surface morphological and structural features of the biomineral structure were studied are marked as segments: (1) inner shell surface, (2) inner shell surface and cross section, and (3) outer edge of the inner shell surface. (B) XRD patterns of the Noah’s Ark shell: (1) powdered sample and (2) native (oriented sample) of the inner shell surface (segment 2 in Figure 1A). (C) Thermogravimetric analysis, TGA (solid line) with differential thermal analysis, DTA (dashed line) (1), total ion current curve for the Noah’s Ark shell (2), and FTIR data from the insoluble organic matrix (IOM) from the Noah’s Ark shell (3).

The surface topography of the inner shell (Figure 1A, segment 2) was determined by atomic force microscopy (AFM) using a MultiMode Probe Microscope with the NanoScope IIIa controller and a “J” scanner with a vertical engagement (JV) of 125 μm (Bruker, Billerica, MA, USA). Imaging was operated in the “Contact mode” using a silicon tip (NP, Bruker, nominal frequency 18 kHz, nominal spring constant of 0.06 N/m) as well as the “Tapping mode” using a silicon tip (TESP, Bruker, nominal frequency 320 kHz, nominal spring constant of 42 N/m) under ambient conditions in the air. The linear scanning rate was optimized between 1.0 and 2.0 Hz at a scan angle of 0°. Images were processed and analyzed utilizing offline AFM NanoScope analysis software, version 1.7.

#### 2.3.2 Determination of Organic Matter Content and FTIR Analysis of the IOM

The organic-matter content in the Noah’s Ark shell was determined by thermogravimetric analysis (TGA) and differential thermal analysis using a NETZSCH Jupiter 449 simultaneous thermal analysis instrument coupled with a mass spectrometer (nano-LC–MS; NETZSCH QMS 403C Aëolos quadrupole). The analysis was performed in the air from 40 to 1000 °C with a heating rate of 10 °C/min using an Al_2_O_3_ crucible with a lid. The evolution of H_2_O and CO_2_ was monitored by *m*/*z* fragments of 18 and 17 and *m*/*z* fragments of 44 and 28, respectively.

Fourier transform infrared spectroscopy (FTIR) was used to characterize the IOM. FTIR spectra of obtained samples were recorded on a NICOLET 320 FTIR Spectrophotometer (Nicodom, Czech Republic) from 600 to 4000 cm^–1^ at room temperature with a uniform resolution of 2 cm^–1^ and 64 scans. For spectral analysis, SpectraGryph software was used.^32^

#### 2.3.3 Extraction and SDS-PAGE Analysis of the Proteins from the Noah’s Ark Shell

The Noah’s Ark shell was prepared for SOM analysis by mechanical grinding in liquid nitrogen. The protein extraction was performed by the method described in our previous work.^4^ In brief, crushed specimens were soaked overnight in 5% (m/v) NaOH, afterward demineralized at 4 °C with a cold diluted acetic acid solution (5% (v/v)) in the presence of protease inhibitor phenylmethylsulfonyl fluoride (Sigma-Aldrich, USA) and sodium azide (Sigma-Aldrich, USA) until the pH reached a value of 4, and centrifuged at 4000*g* before dialysis using cellulose membranes 12–14 kDa (Sigma-Aldrich, USA). The obtained solution was centrifuged at 8000*g* for 60 min, and the supernatant was freeze-dried using a FreeZone 2.5 (Labconco, USA). The resulting powder was dissolved in the buffer (10 mM Tris, pH 8.0) and desalted on a PD10 Sephadex G-25 column (Pharmacia, EU), and the protein concentrations were determined according to the standard Bradford method.^33^

Protein mixtures were analyzed by one-dimensional polyacrylamide gel electrophoresis (PAGE) in the presence of detergent sodium dodecyl sulfate (SDS-PAGE) under denaturing conditions on a 12.5% acrylamide gel^34^ and by two-dimensional gel electrophoresis (2DE). Isoelectric focusing was performed as described by Berkelman and Stenstedt^35^ on an 18 cm IPG strip, pH 3–10. The second dimension SDS-PAGE was performed on a 15% acrylamide gel (20 × 19 × 1.5 mm) using Bio-Rad equipment (Hercules, USA). The proteins in the gels were visualized using Coomassie brilliant blue R-250 dye (Sigma-Aldrich, USA).

#### 2.3.4 N-Terminal Sequencing by Edman Degradation

Proteins were electrotransferred from the 2DE polyacrylamide gel to a polyvinylidene difluoride (PVDF) membrane using Bio-Rad blotting equipment and a transfer buffer supplemented with 10% (m/v) SDS to improve the protein extraction. The proteins on the PVDF membrane were directly N-terminally sequenced by automated Edman degradation on a Procise 492A protein-sequencing system (Applied Biosystems, Foster City, USA).

## 3 Results and Discussion

### 3.1 Formation, Structural, and Morphological Properties of the Noah’s Ark Shell—the Significance of the Colloidally Mediated Mechanism

Besides the well-studied crossed-lamellar aragonite structures of the Noah’s Ark shell,^24,27,28^ the inner-growth surface, which could be important for determining the submicron growth path of this species, has been somewhat neglected in the existing literature. It should be considered that the inner surface of this shell is composed of different functional biomineral structures and it can be assumed that their formation and morphogenesis is based on a nonclassical and colloidally mediated mechanism. A detailed study of the morphological features of these structures may contribute to a better understanding of the basic processes of aragonite formation in this organism.

As expected, XRD data of the pulverized samples showed that the biomineral structure of the Noah’s Ark shell is entirely composed of orthorhombic aragonite (ICDD No. 01–76–0606) (Figure 1B, pattern 1) which has been previously documented.^7,29^ However, XRD analysis of the native inner surface of the shell (Figure 1B, pattern 2) revealed an oriented arrangement of the crystal planes of orthorhombic symmetry. The three large, broadened diffraction peaks of the orthorhombic pinacoid (002), the orthorhombic prism (012), and the orthorhombic bipyramid (312) are the most pronounced.

**Figure 2:**
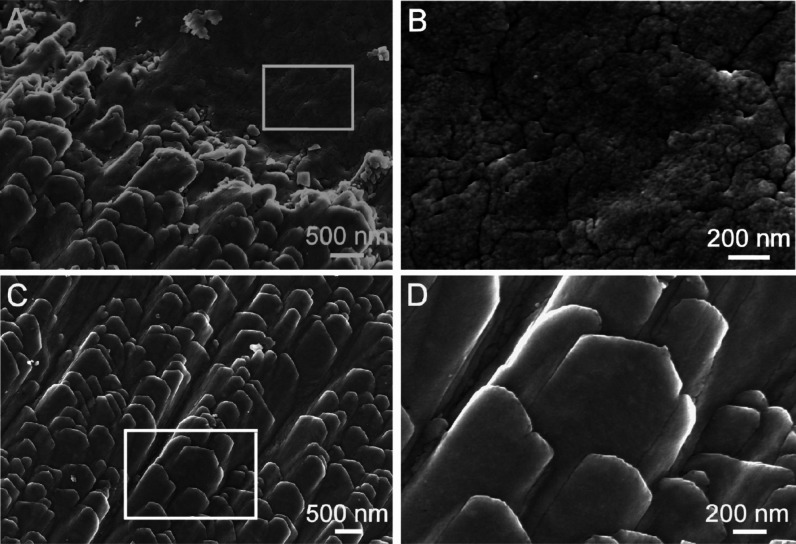
FESEM micrographs of the Noah’s Ark shell. (A,C) Inner growth surface with two details marked under rectangles. (B) Magnification of the rectangle area in (A) is showing a nanogranular structure. (D) Magnification of the rectangle area in (C) is showing elongated morphologically well-developed structures with orthorhombic symmetry.

**Figure 3:**
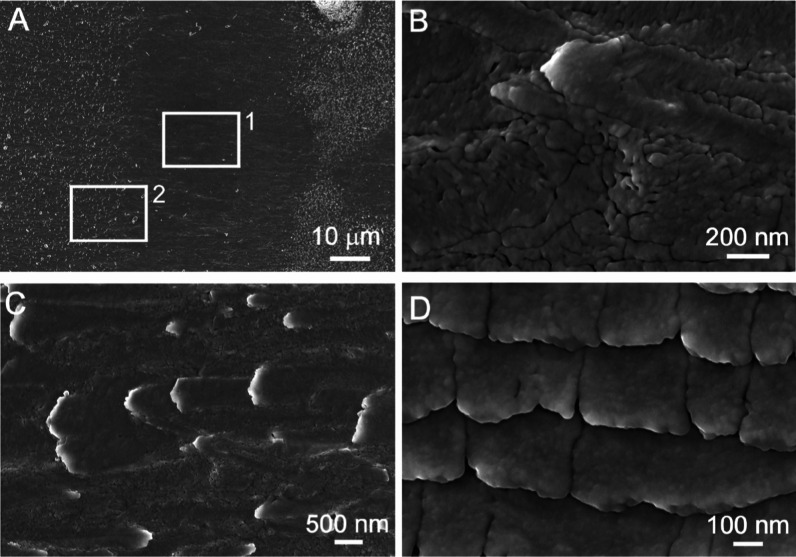
Details of the inner growth surface of the Noah’s Ark shell obtained by FESEM. (A) Detail of segment 3 in Figure 1A. (B) Magnification of the rectangle area 1 in (A) is showing an irregular nanogranular structure. (C) Magnification of rectangle area 2 in (A) is showing an early developmental stage of elongated shapes with orthorhombic symmetry. (D) Developed shapes are showing a nanogranular surface.

This implies that there is orientational growth of the aragonite crystal planes on the inner surface of the shell. The FESEM results obtained by visualizing the same surface support this finding. Several morphological forms occur on the inner surface of the shell, from the nanogranular surfaces to the idiomorphically developed crystals of the orthorhombic habit (Figures 2 and 3). The obvious question was what is the fundamental mechanism responsible for the formation of these different structures on the inner surface of the Noah’s Ark shell?

Numerous studies have shown that, in most cases, nonclassical crystallization through aggregation-based crystal growth, the formation of aligned shapes and mesocrystals are identified as important mechanisms in the formation of biomineral structures.^5,13−16,19,20^ In our previous studies, we have shown that oriented aggregation and subsequent coalescence processes of primarily formed aragonite nanoparticles are the main pathway in the formation of the complex aragonite biomineral structures in *S. officinalis*(4) and *C. caespitosa*.^10^ The organization and morphology of aragonite on the inner growth surface of the Noah’s Ark shell are different from these biomineral structures, and it is reasonable to assume that compared to the formation and morphogenesis of aragonite in these species, the growth pathway in the Noah’s Ark shell appears to be somewhat different. Marin et al. presented how diverse shell microstructures may be, from prismatic to spherulitic to laminar, with different types and subtypes.^24^ Crossed-lamellar microstructures are the most common ones, also present in the Noah’s Ark shell.

It is reasonable to assume that different morphological structures are formed by morphogenesis in multistage processes, which can be followed by analyzing the morphological features of the biomineral structures on the inner growth surface of the Noah’s Ark shell.

It is generally thought that the formation of the aragonite phase in biomineral structures proceeds through several stages.^4,6,11,19,26,37^ According to previous findings obtained in other mollusk species, the first stage in the formation of Noah’s Ark shell biomineral structures should involve the organically mediated formation of the transitional and metastable amorphous phase—amorphous calcium carbonate (ACC). Although this phase is not determined in this study, numerous studies have shown that many organisms employ a strategy to convert ACC to more stable forms of carbonates by manipulating the structural and morphological properties of their biomineral structures.^1,11,36−38^ This phase then converts into the thermodynamically more stable, nanoscale, and well-crystallized aragonite, which forms nanogranular surfaces visible in some parts of the internal growth structures (Figures 2A,B and 3A,B). Nanosolids are known to play a significant role in biomineralization processes, either in the form of nanocluster precursors or nanoparticles.^39^ Several studies have supported this statement and have reported and discussed the formation of nanoscale aragonite structures.^10,11,19,36^ Moreover, aragonite has been shown to exhibit oriented growth of its biomineral forms very frequently in many biomineral structures.^4,24,40^

**Figure 4:**
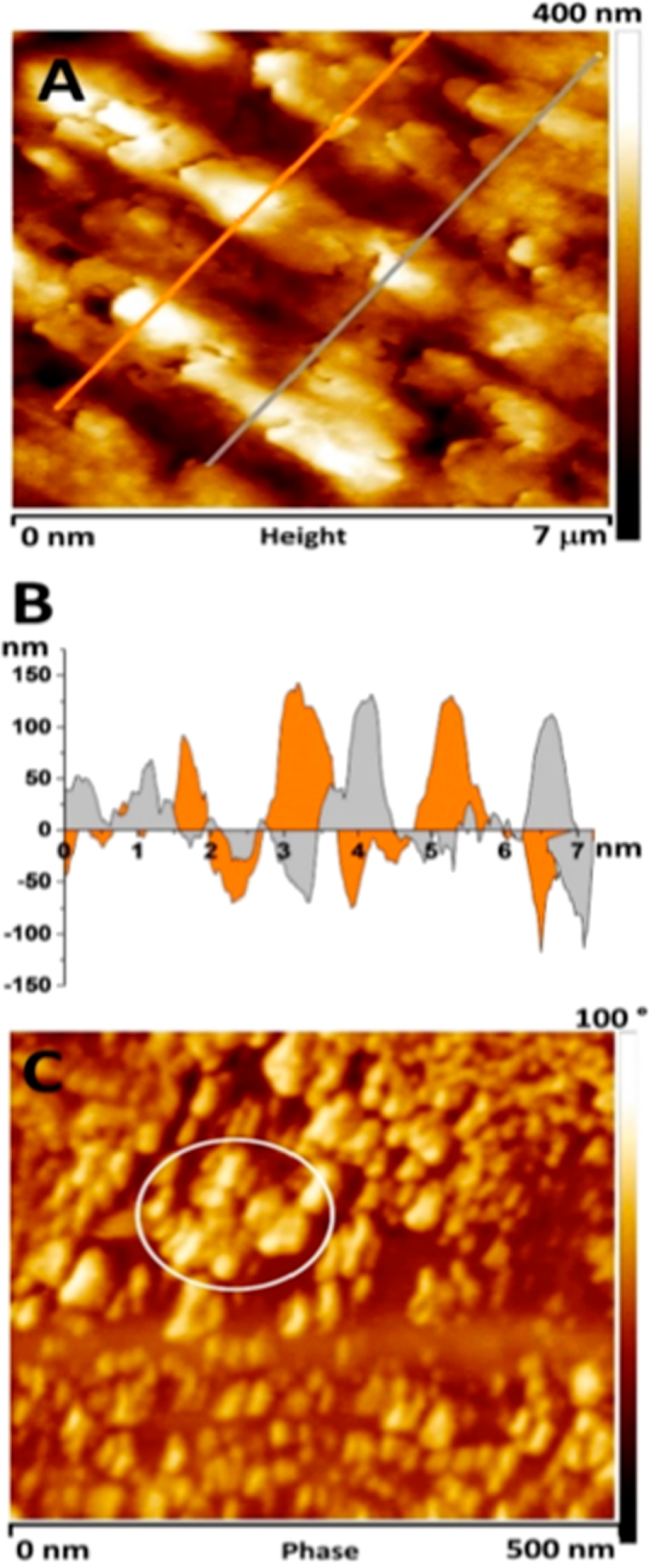
AFM topographic data of the native inner surfaces of the Noah’s Ark shell. (A) Data measured in the contact mode (rectangle area 2 in Figure 3A) with marked positions where profile sections were measured. (B) Profile sections of that surface. (C) The inner surface (measured in tapping mode) corresponds to the nanogranular surface, marked as area 1 in Figure 3A.

The inner shell surface was additionally investigated by AFM to get better insight into its nanostructures. The AFM images in Figure 4 show the surface topography of segment 3, which is consistent with the FESEM images in Figure 3. The AFM image in Figure 4A shows the surface topography of the upper part of the elongated shapes marked with a white square 2 in Figure 3A. The profile section data of this area show that their height ranges from 50 to 150 nm (Figure 4B). Additional characterization of the inner nanogranular surfaces and their phase data of the region marked with a white square 1 in Figure 3A was performed with AFM in tapping mode (Figure 4C). Differences in the phases between harder and softer materials may indicate different nanocompositions of the two domains; harder spherulitic granules surrounded by softer organic matter produce a different phase, which can be seen in a darker color in Figure 4C.^39,41^ It can be seen that the small and almost spherical particles have a size of 30 to 50 nm. They appear mainly as single grains in the organic matrix, but areas where they are aggregated can also be seen (the area marked with a circle in Figure 4C). However, they cannot be considered as oriented structures or aligned nanoparticle shapes, consistent with the FESEM micrographs in Figures 2B and 3B.

It can also be seen that much larger, micrometer-sized, elongated structures appear on the inner surface. They show the oriented and parallel growth and shape typical of orthorhombic symmetry (Figure 2C,D). The question is what is the basic formation mechanism of these structures and how they are related to nanogranular surfaces? Recently, Rodriguez-Navarro et al.^19^ presented a general review of the current knowledge about the nonclassical crystallization in the formation of some biomineral structures. Among other processes, they also mention the role of dissolution–recrystallization processes, which could be one of the mechanisms in the formation of some biomineral structures.

We have proposed that during the second phase, the dissolution–recrystallization process is the main pathway in the formation of well-defined micrometer-sized orthorhombic crystal forms of aragonite in the biomineral structure of the Noah’s Ark shell. It is governed by the minimization of surface energy,^19,42−44^ where the initially formed and thermodynamically less stable aragonite nanoparticles transform into a well-crystallized form of aragonite with micron-sized dimensions through their dissolution and recrystallization. This leads to the development of different aragonite structures on the inner growth surface of the Noah’s Ark shell. Indeed, Figure 3C clearly shows the early morphological transformation of nanogranular textures into the well-defined micrometer-sized aragonite crystals with orthorhombic symmetry. However, this is primarily a morphological transformation that is not accompanied by a change in the crystal structure and a transformation into other polymorphic forms of anhydrous calcium carbonate. In fact, the presence of calcite and vaterite in the structure of the shell was not detected (Figure 1B).

**Figure 5:**
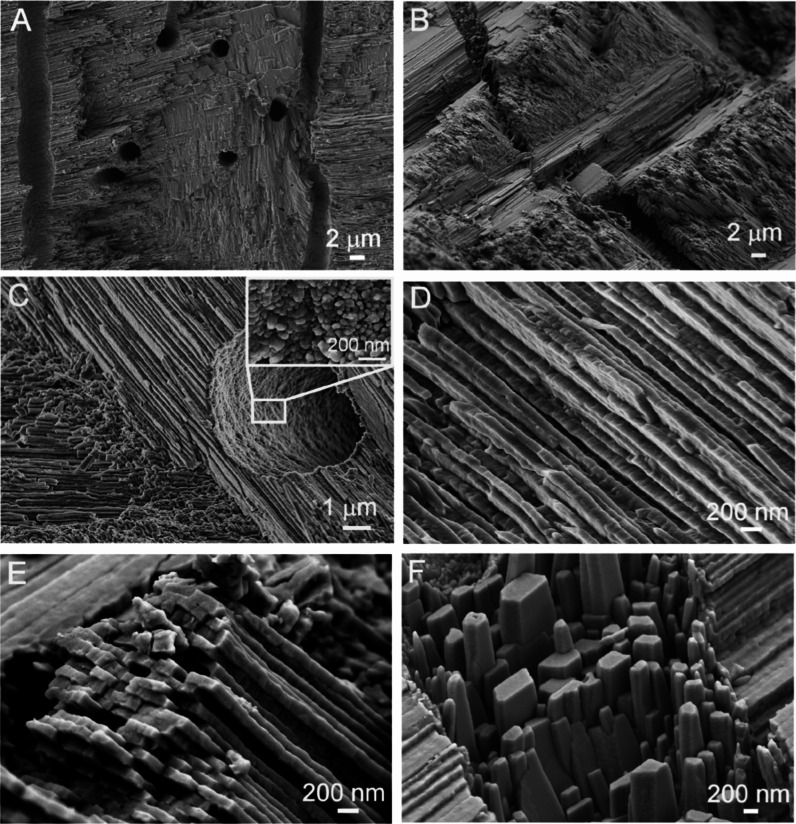
Details of the Noah’s Ark shell in cross section (segment 2 in Figure 1A) obtained by FESEM. (A,B) CCL structure with parallel tubules protruding in multiple directions. (C) Magnification of the stack with a visible tubule opening with a highlighted nanostructure of the tubule surface (inset). (D,E) Details of the structure built from lamellae with nanodimensions. (F) Oriented growth of the idiomorphic aragonite crystal with pronounced orthorhombic symmetry.

Although the CCL structure visible in the cross section of the Noah’s Ark shell (segment 2) (Figure 5A,B) is well-described in the literature,^27^ there are nevertheless some observations made in this study that need to be emphasized. The thickness of these lamellae is about 100–400 nm. Their different orientation and linkage lead to the formation of a distinct CCL morphology of the shell (Figure 5C–E). This type of arrangement is not exclusive to arcids and is also found in many other bivalves, usually as an inner layer surrounded by a crossed lamellar structure, as is the case in the Noah’s Ark shell.^18,29,45−47^ What has not yet been documented in the biomineral structure of the Noah’s Ark shell is the presence of well-developed aragonite crystals, which have the shape of an orthorhombic pinacoid and prism (Figure 5F). One of the interesting observations is the remaining nanogranular structure within the tube of the shell, indicating colloid-mediated pathways of crystallization on the nanoscale (Figure 5C). According to a previous study,^41^ the growth of the Noah’s Ark shell with lamellar aragonite structure is divided into two phases. The first phase involves the formation of ACC precursors on the organo-mineral surfaces. The second phase involves the aggregation and coalescence of the primary formed spheroidal subunits during the growth of the crystal front. The results obtained in this study contradict the proposed mechanism. We are convinced that the formation of well-developed, micrometer-sized orthorhombic aragonite crystals (Figure 5F) during the second phase of morphogenesis cannot be the result of aggregation processes. Rather, it is a result of the previously described model based on the dissolution–recrystallization processes.

### 3.2 Characterization of the Organic Matrix

To estimate the percentage of organic matter embedded in the biomineral structure, the powder of the shell was analyzed by TGA and DSC (Figure 1C). The results obtained showed that the content of organic matrix and water in the Noah’s Ark shell is 2.6% of the total mass. This relatively low percentage of water and the organic matrix is consistent with related studies showing that the organic compounds in similar biomineral structures account for about 2.2–2.6% of the total mass.^48−50^ It has been reported that about 1% of the mass loss is due to water structurally associated with the hydrated matrix proteins associated with the mineral components.^2,51^ Therefore, the content of organic materials in the Noah’s Ark shell is estimated to be 1.5% of the total mass. As previously reported, the stability of the biomineral structure decreases by an astonishing 95% when the organic component is removed from the shell, as the organic matter acts as a viscoelastic glue between the mineral layers, increasing their elasticity.^52,53^

To investigate the basic features of an organic matrix of the biomineral structure of the Noah’s Ark shell, both IOM and SOM were isolated. The IOM was characterized by FTIR spectroscopy. The FTIR spectrum of the IOM (Figure 1C) is characterized by the O–H stretching vibrations in the range between 3500 and 3200 cm^–1^ and the C–O–C and C–O stretching vibrations in the range of 1200–950 cm^–1^. Dominant vibrations at 1630 and 1035 cm^–1^ indicate the presence of the chitin component.^54,55^ This result is similar to previous results describing the properties of the IOM from the biomineral structure of cuttlebone.^56,57^ It is evident that the IOM has a strong preference for chitin and its derivatives, the well-known polymer that normally serves as a scaffold for initial biomineral attachment and growth.^57^

Previous studies have shown that the predominant organic compounds involved in biomineralization are acidic proteins.^1,25,58^ Nevertheless, also basic proteins, for example, N25 from the Akoya pearl oyster (*Pinctada fucata*), was found to modify calcium carbonate morphology and shell biomineralization.^59^ Proteins isolated from the Noah’s Ark shell appeared on 1D SDS-PAGE as bands in the range of 25–50 kDa (Figure S2A). 2DE analysis showed only the most abundant proteins of about 43 kDa that were predominantly acidic with isoelectric points (pIs) in the pH range 3–5 (Figure S2B). A series of protein spots with approximately the same molecular mass but focused at different pH values (spots 1–12 in Figure S1B) most likely represent different post-translationally modified forms (e.g., glycoforms and phophoforms) of the same protein.

Proteins associated with biomineralization in mollusks have been intensively studied,^18,60^ but the composition of the organic matrix of many marine invertebrates remains unknown. These proteins have been shown to be diverse and highly species-specific with no clear homology.^24^ What they have in common is that they are often post-translationally modified, for example, glycosylated and phosphorylated.^24,58^ Therefore, they are unlikely to be easily identified when searching sequence databases.

**Figure 6:**
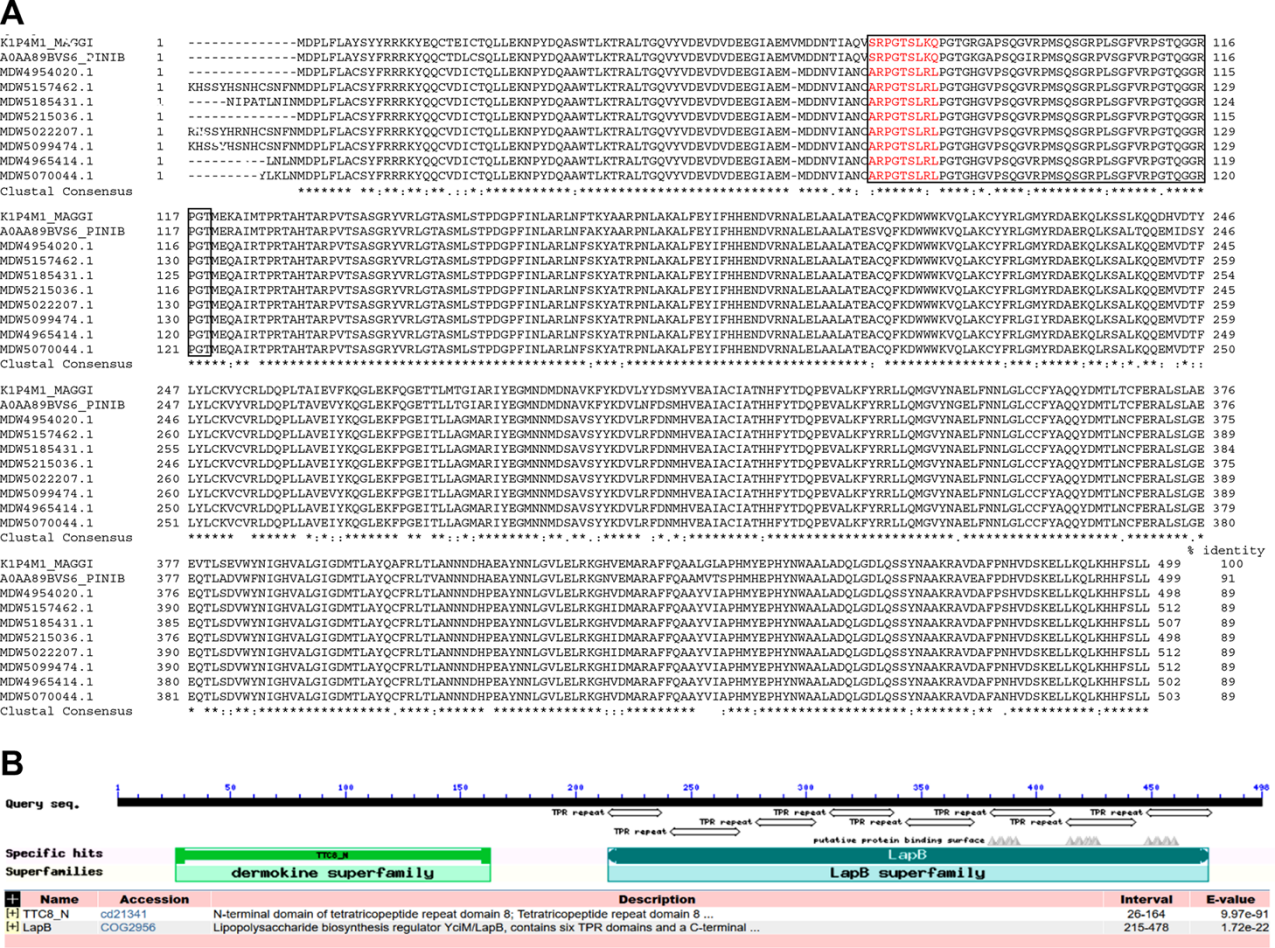
Alignment of the primary structures and structural analysis of proteins with homology to the N-terminal sequence of the *Arca noae* protein. (A) We used Clustal Omega^65^ for multiple sequence alignment of tetratricopeptide repeat (TPR) protein 8 from the following mussels: *Crassostrea* (*Magallana*) *gigas* (K1P4M1_MAGGI), *Pinctada martensii* (*imbricata*) (A0AA89BVS6_PINIB), *Fusconaia askewi* (MDW4954020.1), *Tritogonia verrucosa* (MDW5157462.1), *Toxolasma texasiensis* (MDW5185431.1), *Quadrula quadrula* (MDW5215036.1), *Megalonaias nervosa* (MDW5022207.1), *Ambleminae* gen. n. sp. n CS-2023 (MDW5099474.1), *Truncilla macrodon* (MDW4965414.1), and *Potamilus inflatus* (MDW5070044.1). The sequence region with similarity to the *Arca noae* sequence ARPGPGLRL is shown in red. The disordered region predicted by UniProt’s Automatic Annotation is marked with a rectangle. In the “Clustal Consensus” row, an asterisk (*) denotes identical amino acids in all aligned sequences, a colon (:) denotes very similar amino acid residues in the aligned sequences (conservative substitutions), and a dot (·) denotes somewhat similar amino acid residues in the aligned sequences (semiconservative substitutions). (B) The NCBI conserved domain search categorized the proteins into the dermokine and LapB/TPR-like helical domain superfamily. The conserved domains of dermokines contribute to the maintenance of proper folding and stability of proteins and mediate protein-protein interactions. Consistent with the potential molecular function of TPR proteins 8, three putative protein binding sites were recognized in the C-terminal part.

To obtain information about the protein structure, we used N-terminal amino acid sequencing. The most intensive protein spots on the 2DE gel with an apparent molecular mass of 43 kDa and different pIs (Figure S1B), were electroblotted onto a PVDF membrane and sequenced. In all cases, the same sequence XARPGPGLRL was obtained, where X stands for an unidentified amino acid residue. As already mentioned, SOM proteins in mollusks are frequently post-translationally modified,^24,58^ so our interpretation is that X corresponds to a post-translationally modified amino acid residue. BLAST analysis of the obtained sequence using the nonredundant database “Transcriptome shot-gun assembly proteins” and taxa Mollusca (taxid:6447) revealed the highest similarity with the sequence ARPGTSLRL found in the N-terminal part of tetratricopeptide repeat (TPR) protein 8 from different freshwater mussels (protein identification numbers: MDW5022207.1, MDW5157462.1, MDW5099474.1, MDW5185431.1, MDW5070044.1, MDW4965414.1, MDW4954020.1, MDW5215036.1). TPR protein 8 was found also in Atlantic pearl-oyster (*Pinctada martensii* (*imbricata*), A0AA89BVS6_PINIB) and Pacific oyster (*Crassostrea* (*Magallana*) *gigas*, K1P4M1_MAGGI)^61^ having a similar sequence SRPGTSLKQ in the corresponding region (Figure 6A). The common structural features of these proteins are a disordered region in the N-terminal part (according to UniProt’s Automatic Annotation) and eight TPR repeats characteristic of the tetratricopeptide-like helical domain superfamily of proteins (IPR011990), which overlap with the homologous LapB superfamily, as identified by the NCBI Conserved domain search of the sequences (Figure 6B). A study of 39 aragonite-associated proteins from molluscs found that all had predicted disordered regions, suggesting that this trait may drive shell matrix assembly, similar to processes in the vertebrate extracellular matrix.^62^

TPR repeats fold into α-helices, which assemble in the form of superhelices and provide a large solvent-accessible surface area that is well suited for binding large substrates such as proteins and nucleic acids. TPR motifs are crucial in multiprotein complexes and support functions such as protein folding, cell cycle regulation, transcription, and protein transport. For this reason, TPR motifs are found in all kingdoms of life and regulate diverse processes, including organelle targeting, protein import, vesicle fusion, and biomineralization.^63^ For example, MamA, a magnetosome-associated protein containing six TPRs, is involved in biomineralization of iron oxides in magnetotactic bacterium where it guides biomineralization proteins assembly.^64^

## 4 Conclusions

Structural and morphological features as well as the formation of the biomineral aragonite structure of the Noah’s Ark shell were studied. Based on the obtained results, it was found that the main mechanism of formation of its nanogranular surface and μm-sized orthorhombic aragonite structures is determined by the nonclassical crystal growth mechanism, which takes into account the colloidally mediated processes involving the initial particle–particle interaction of the nanosized and metastable precursor aragonite phase and its dissolution and recrystallization processes.

For the first time, SOM-specific proteins from the biomineral structure of the Noah’s Ark shell have been analyzed. The estimated amount of SOM, 1.5% of the total biomineral structure, is as in other shells. The aragonite-associated SOM contains several proteins potentially involved in biomineral assembly. Most of them are acidic and apparently post-translationally modified. The partial sequence of 43 kDa proteins shares a similarity to multidomain TPR protein 8 from different mussels. By analogy to the bacterial TPR protein, we suggest that the TPR-containing proteins are involved in the biomineralization process in *Arca noae* and related mussels as well. Future research will be more focused on the identification of additional proteins potentially involved in biomineralization and revealing their fine functions in this complex process.

## Dedication

We dedicate this paper to the memory of the late Dr. Ivan Sondi, our dear colleague and friend, who was an expert in biomineral structures and a leader of this project. Dr. Ivan Sondi passed away on March 9, 2024.
