# Supplementary material for: Morphogenesis of Aragonite Biomineral Structures by the Nonclassical Colloidal Crystal Growth Mechanism Revisited on the Nanoscale: The Noah’s Ark Shell (Arca noae, L.) Case Study
Source: ACS Biomater Sci Eng. 2025 Jan 29;11(2):866–74. doi: 10.1021/acsbiomaterials.4c01420 (PMC11815636; doi:10.1021/acsbiomaterials.4c01420)
Supplement: Supplementary file 1 — ab4c01420_si_001.pdf [file ab4c01420_si_001.pdf]

SUPPORTING INFORMATION

Morphogenesis of aragonite biomineral structures by the nonclassical colloidal crystal growth mechanism revisited on thenanoscale: the Noah's Ark shell (*Arca noae*, L.) case study

Ivan Sondi[a], Adrijana Leonardi[b], Igor Križaj[b], Saša Kazazić[c], Branka Salopek-Sondi[d], Srečo D. Škapin*[e]

[a] Faculty of Mining, Geology and Petroleum Engineering, 10000 Zagreb, Croatia
[b] Department of Molecular and Biomedical Sciences, Jožef Stefan Institute, 1000 Ljubljana, Slovenia
[c] Division of Physical Chemistry, Ruđer Bošković Institute, 10000 Zagreb, Croatia
[d] Division of Molecular Biology, Ruđer Bošković Institute, 10000 Zagreb, Croatia
[e] Advanced Materials Department, Jožef Stefan Institute, 1000 Ljubljana, Slovenia

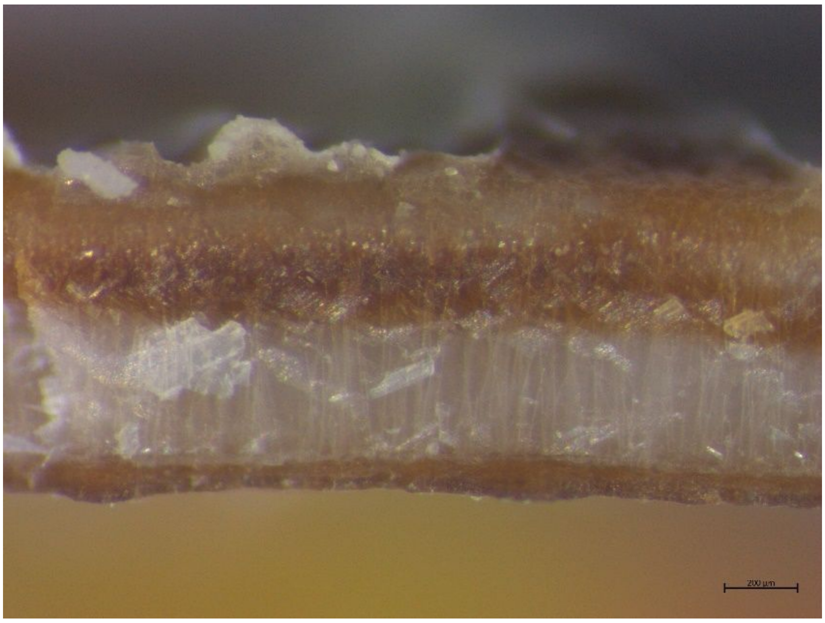

Figure S1. Picture of Noah's Ark shell fracture, taken with stereo microscope.

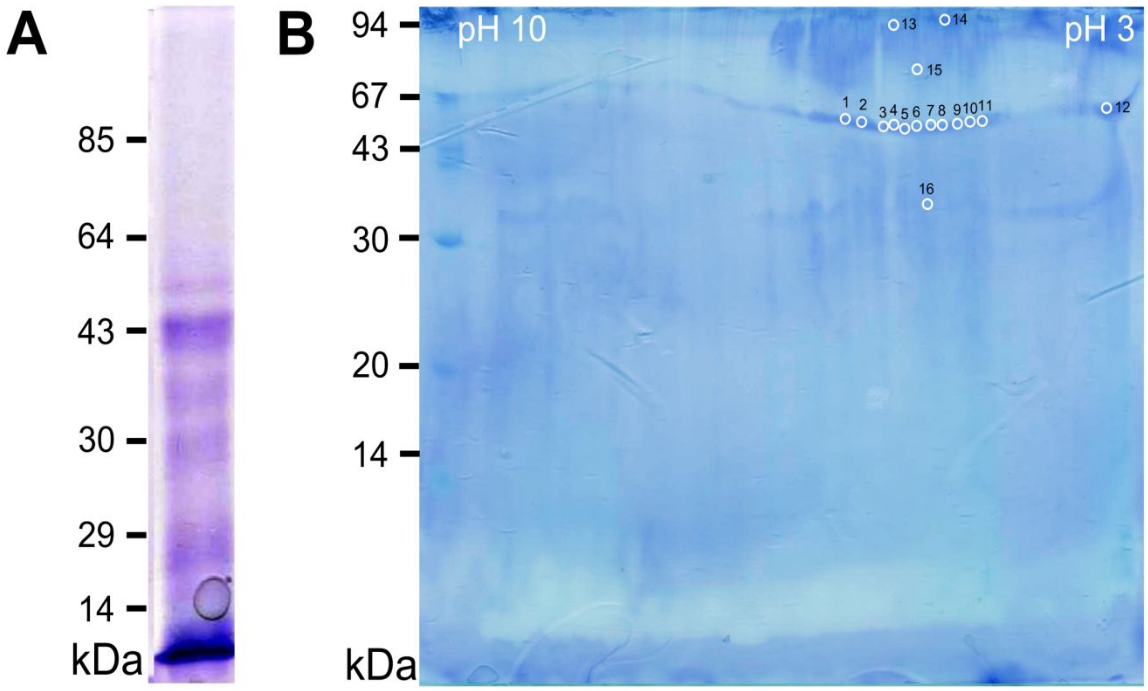

Figure S2. Soluble organic matrix proteins from the Noah's Ark shell analyzed using: (A) 1D SDS-PAGE on the 12.5% gel, and (B) 2DE on the 15% gel. Positions of the molecular mass standards and the pH range are indicated. Proteins were stained with Coomassie Brilliant Blue R-250. Following 2DE, proteins were electro-transferred from the gel onto a PVDF membrane and the indicated spots were analyzed by automated Edman sequencing.
